# Toxicity Effects of Fine Particulate Matter (PM_2.5_) from Incomplete Solid Fuel Burning in *Caenorhabditis elegans*

**DOI:** 10.3390/toxics14070597

**Published:** 2026-07-08

**Authors:** Zhenyu Lu, Bingbo Huang, Xiaoming Liu, Wankang Chen, Xiaoyu Cai, Mindong Chen

**Affiliations:** 1 Collaborative Innovation Center of Atmospheric Environment and Equipment Technology, Jiangsu Key Laboratory of Atmospheric Environment Monitoring and Pollution Control, School of Environmental Science and Engineering, Nanjing University of Information Science & Technology, Nanjing 210044, China; nuistlzy@163.com (Z.L.); 13182805780@163.com (B.H.); caixiaoyu8410@163.com (X.C.); 2 Jiangsu Environmental Engineering Technology Co., Ltd., Nanjing 210044, China; 3 School of Environment, Southern University of Science and Technology, Shenzhen 518055, China; 12531399@mail.sustech.edu.cn

**Keywords:** *C. elegans*, PM_2.5_, biomass burning, ROS, PAHs

## Abstract

Although the health risks associated with the use of biomass fuels have received widespread attention, there has been insufficient detailed research conducted on the toxic effects and toxicity generation mechanisms of PM_2.5_ produced by the use of different sources of solid organic fuels. In this study, the synchronized L4-stage *Caenorhabditis elegans* (*C. elegans*) were exposed to the suspensions of the PM_2.5_ samples collected from incomplete combustion products of rice straw, wheat straw, peanut straw, rapeseed straw and the branch of poplar and paulownia. Body length, the number of fertilized eggs, accumulation of lipofuscin, and levels of reactive oxygen species (ROSs) were measured to characterize developmental toxicity, reproductive toxicity, intestinal damage, and oxidative stress. The types and mass proportions of organic carbon (OC), elemental carbon (EC), water-soluble inorganic ions, and polycyclic aromatic hydrocarbons (PAHs) in PM_2.5_ were determined. The results show that PM_2.5_ generated from the combustion of the straw of oilseed crops such as peanuts and rapeseed has the most severe toxic effects on *C. elegans*. The toxicological mechanism was mainly mediated by severe oxidative stress and excessive generation of ROSs. The chemical characteristics of PM_2.5_ have strong source-specificity, and its toxic effects are closely related to the high content of lipid-soluble PAHs in PM_2.5_ from oilseed crop sources.

## 1. Introduction

Incomplete combustion of solid fuels is recognized as a major source of fine particulate matter (PM_2.5_) in the global atmosphere [1,2]. PM_2.5_ emitted from residential solid fuel combustion has been reported to exhibit the highest toxicity among anthropogenic sources. These particles can penetrate biological barriers and exert adverse effects on organisms, posing significant risks to the ecological environment and human health. Chemical components of PM_2.5_ from different solid fuel sources are highly heterogeneous, which directly determines differences in toxicological effects [3,4]. China is one of the largest consumers of biomass energy worldwide, with more than 600 million rural residents relying on solid biomass fuels for daily energy needs [5]. Agricultural residues and firewood account for over 90% of the total biomass consumed in rural China, with annual straw production exceeding 800 million tons [6].

Eastern China has been identified as one of the most severely polluted regions by incomplete combustion emissions in China, characterized by intensive agricultural activities and high population density [7]. Rice straw and wheat straw are the most abundant crops in this region [8,9]. Peanut and rapeseed, the primary oilseed crops in eastern China, produce more than 50 million tons and 30 million tons of straw annually, respectively [10]. Paulownia and poplar, both widely planted fast-growing hardwood species for timber production, generate approximately 10 million tons of pruning and processing waste annually. A considerable portion of the above crop straws were treated by open burning, and different biomass showed different combustion characteristics, resulting in potentially different emission characteristics and toxicological characteristics [11]. These straws cover the most highly produced and the most extensive types of domestic combustion in eastern China, and can effectively represent typical local biomass combustion sources. The emissions of these local dominant biomass fuels have a profound impact on regional air quality and public health, so it is particularly critical to study the toxicity of particulate matter produced by biomass burning [12].

However, there is currently a lack of systematic research on the chemical properties of PM_2.5_ generated from the incomplete combustion of various common residential solid fuels and its toxic effects on model organisms. Most previous studies have focused on the products of a single fuel or PM_2.5_ in the actual atmospheric environment, such as specific crop straw or firewood [4,13,14,15]. These studies independently reported the toxicity profiles of single-fuel-derived PM_2.5_, but included no comparisons across diverse fuel types, thus failing to clarify the influence of fuel properties on toxicity magnitude and key toxic constituents. This has led to the unclear identification of the key components and potential mechanisms that affect the toxicity of the incomplete combustion products of different solid fuels.

To clarify the toxic effects of PM_2.5_ generated through biomass burning, appropriate model organisms are required for systematic toxicological evaluation. *C. elegans* has been widely employed as a model animal in toxicological research due to its advantages of a short life cycle, simple structure, high sensitivity to environmental toxicants, and conserved biological pathways with higher organisms [16,17,18]. In previous studies, *C. elegans* has been observed to be sensitive to PM_2.5_ exposure, with significant adverse effects on its growth, reproduction and intestinal health [19,20].

In this study, rice straw, wheat straw, peanut straw, and rapeseed straw, as well as branches of poplar and paulownia, were selected as representative materials for biomass fuels as the research subjects. Based on the sources of these fuels and the differences in the uses of the corresponding agricultural and forestry crops, they are classified as straw of grain crops (rice straw, wheat straw), straw of oilseed crops (peanut straw, rapeseed straw) and firewood (branches of poplar and paulownia). PM_2.5_ samples were collected from incomplete combustion of the selected fuels, and comprehensive chemical component analysis was performed to clarify source-dependent differences in chemical profiles. The toxicological effects of PM_2.5_ on *C. elegans* were evaluated by detecting developmental inhibition, reproductive damage, intestinal injury, and oxidative stress. The underlying toxicological mechanisms were explored based on correlations between chemical components and toxicological endpoints. This study aims to provide a scientific basis for risk assessment of PM_2.5_ from incomplete solid fuel burning and formulation of relevant environmental protection strategies.

## 2. Materials and Methods

### 2.1. PM_2.5_ Collection and Preparation

The collected biomass fuels were first dried in an oven to remove moisture. The materials were then processed into blocks measuring approximately 10~15 cm in length. For each type of biomass fuel, 300 g were weighed and sealed in clean airtight bags, which were then stored in a dry environment for subsequent experiments.

The combustion experiment was conducted in a simulated combustion system. Approximately 300 g of biomass fuel was placed in the combustion furnace and ignited each time. Sampling of particulate matter began immediately when smoke appeared in the vertical flue. Sampling ceased when there was no longer any obvious flame in the furnace and no obvious smoke was emitted from the flue. The combustion time of crop straw was approximately 5 to 15 min, while that of firewood was approximately 10 to 20 min.

Particulate matter was collected using a HY-206 type dilution channel particulate membrane sampler. In the dilution channel, the hot flue gas was diluted by clean air, cooled to the ambient temperature, and then introduced into the sampling chamber. PM_2.5_ particles were captured on 47 mm quartz filter membranes. Before sampling, the quartz filter membranes were baked in a muffle furnace at 500 °C for more than 5 h to remove moisture and volatile organic compounds, placed in a dryer (25 °C, 35% relative humidity) for 24 h, and then weighed to obtain the blank membrane mass. The particulate matter in the smoke was collected onto four filter membranes labeled A, B, C, and D through four parallel pipelines. The total sampling flow rate was approximately 40 L/min, and the flow rates of the four parallel pipelines were roughly the same during sampling. After sampling, the filter membranes were placed in the dryer for 24 h and weighed to obtain the mass of the collected particulate matter. The filter membranes were wrapped in aluminum foil and stored at −20 °C for further chemical and toxicological analysis.

### 2.2. Chemical Component Analysis of PM_2.5_

The contents of organic carbon (OC) and elemental carbon (EC) in the PM_2.5_ samples were determined using a thermal/optical carbon analyzer (Sunset Laboratory Inc., Tigard, OR, USA, Model 4). A designated area of the filter was punched and placed into the front furnace of the analyzer. The sample was progressively heated to 850 °C in a pure helium (He) atmosphere to thermally desorb organic compounds, followed by a second heating stage in a He/O_2_ (2%) mixed atmosphere. The evolved carbonaceous compounds were subsequently oxidized to CO_2_ in a manganese dioxide (MnO_2_) oven and quantified using a non-dispersive infrared (NDIR) detector. Sucrose was utilized as the standard for instrument calibration, with a relative deviation strictly controlled below 5%.

Water-soluble inorganic ions were quantified using Dionex ICS-2000 (for anions: Cl^−^, NO_3_^−^, SO_4_^2−^ and ICS-3000; for cations: NH_4_^+^, Na^+^, K^+^, Mg^2+^, Ca^2+^ ion chromatographs). For sample pretreatment, two 8 mm diameter punches from each filter were sectioned into a beaker, and 40 mL of ultrapure water was added. The mixture was ultrasonically extracted in an ice bath for a total of 60 min (two 30 min cycles). After being filtered through PTFE filters with a pore size of 0.22 μm, the extracts were collected in centrifuge tubes and stored at 4 °C. Anion separation was performed using an IonPac AS11-HC analytical column (4 × 250 mm) with a 10 mmol/L KOH eluent. Cation separation utilized an IonPac CS16 column (5 × 250 mm) with a 32 mmol/L methanesulfonic acid (MSA) eluent. Standard curves were established using concentration gradients ranging from 0.05 to 10.0 μg/L, and field blanks were systematically subtracted from the sample results to ensure accuracy.

Sixteen EPA-priority PAHs in the PM_2.5_ samples were determined utilizing a gas chromatograph–mass spectrometer (GC-MS, Agilent 7890B/5977B, Agilent Technologies Inc., Santa Clara, CA, USA). Two filter membrane samples with a diameter of 1 cm were immersed in 5 mL of dichloromethane. The filter membranes were subjected to ultrasonic treatment in an ice bath to extract the aqueous solution samples. The total extraction time was 60 min (divided into three 20 min cycles). The extracts were subsequently filtered through a 0.22 μm PTFE filter, concentrated to a final volume of 1 mL under a gentle stream of high-purity nitrogen, and stored at 4 °C. Chromatographic separation was achieved on an HP-5 MS capillary column (30 m × 0.25 mm × 0.25 μm) utilizing high-purity helium as the carrier gas. The GC oven temperature program was initiated at 50 °C for 1 min, then ramped at a rate of 30 °C/min to 325 °C, where it was held for 10 min. Quantification was strictly performed using the internal standard method operating in selected ion monitoring (SIM) mode.

Specific organic markers, encompassing methoxyphenols and related hydroxyl compounds, were systematically analyzed using high-performance liquid chromatography–mass spectrometry (HPLC-MS). A 2 cm diameter filter punch was spiked with an internal standard mixture (containing phthalic acid-d4, trans-cinnamic acid-d7, and (1R,2S)-cyclobutane-1,2-dicarboxylic acid) and allowed to equilibrate for 30 min to ensure thorough membrane infiltration. The sample was then ultrasonically extracted in an ice bath (strictly maintained below 25 °C) for a total of 60 min (three 20 min cycles). Following filtration through a 0.22 μm PTFE filter, the extract was evaporated to dryness under a nitrogen stream. The residue was carefully reconstituted in 500 μL of a mixture consisting of 0.01% aqueous formic acid and methanol/acetonitrile (4:1, *v*/*v*) in a 75:25 (*v*/*v*) ratio. Chromatographic separation was effectively performed on a Thermo Hypersil C18 column (100 mm × 2.1 mm, 3 μm) maintained at a column temperature of 30 °C with a constant flow rate of 350 μL/min. A gradient elution protocol was employed using 0.01% formic acid in water as Mobile Phase A and methanol/acetonitrile (4:1) as Mobile Phase B.

### 2.3. C. elegans Strains and Exposure Methods

The strain used in this study was wild-type N2, which was provided by the Caenorhabditis Genetics Center (CGC, University of Minnesota, Twin cities, USA). *C. elegans* were maintained in darkness at 20 °C and cultured on nematode growth medium (NGM) plates seeded with Escherichia coliOP50 [21]. Gravid adults were washed into centrifuge tubes using a K-medium buffer, and a prepared bleaching solution was added [22]. The supernatant was subsequently removed, and the released eggs at the bottom were washed with sterile K-medium to eliminate any adverse effects from the lysate. The collected eggs were then placed onto fresh NGM plates and incubated at 20 °C for 48 h to obtain synchronized L4-stage larvae.

PM_2.5_ samples were eluted from quartz filter membranes with K-medium buffer via 30 min ice-bath sonication, and then adjusted to a stock concentration of 1000 μg/L before the experiment. A full procedural blank control was strictly set to eliminate potential interferences from filter membranes and sample processing. Specifically, blank quartz filter membranes from the same batch, which underwent exactly the same pre-treatment as sampling membranes (500 °C baking in muffle furnace for 5 h and 24 h constant-weight drying in a desiccator), were processed following completely identical elution and dilution procedures with the K-medium buffer. The obtained procedural blank solution served as the control group, sharing the exact same solution matrix and full processing workflow with all PM_2.5_ exposure groups, with the only difference being the absence of combustion-derived particulate matter.

Synchronized L4 larvae were transferred to a 12-well plate. A 500 µL volume of the PM_2.5_ suspension was then applied to each well, which also contained an adequate amount of Escherichia coliOP50 as a food source to prevent any restriction on the growth of *C. elegans*. Each concentration group consisted of twenty *C. elegans*, and three independent experimental replicates were performed.

### 2.4. Assay of Fecundity

In this study, the number of fertilized eggs in *C. elegans* after 24 h of acute exposure was determined to assess the effect of PM_2.5_ from incomplete solid fuel burning on the reproductive capacity. Three independent biological replicates were performed for each exposure group, with 20–25 synchronized L4 larvae included in each replicate. Following 24 h of acute exposure, *C. elegans* were collected and washed three times with the K-medium buffer to remove residual PM_2.5_ particles. Each nematode was then transferred onto NGM plates and anesthetized with levamisole solution (60 µM). The number of released eggs per nematode was counted under a stereomicroscope for statistical analysis.

### 2.5. Assay of Body Length

Following exposure, *C. elegans* were collected and washed three times with the K-medium. After washing, *C. elegans* were placed in a constant temperature water bath at 60 °C for 30 min. The appropriate amount of liquid was collected using a pipette gun and placed on a slide. The slide was placed under a microscope, and images were captured using the software Image View 9.0. The body length was calculated by processing the images with the software ImageJ V1.8.0. At least 30 nematodes were measured in each group.

### 2.6. Assay of Lipofuscin

Intestinal autofluorescence caused by lipofuscin deposited in lysosomes can accumulate over time in *C. elegans* that are aging or exposed to toxicants, as previously described. After exposure, *C. elegans* were washed three times and anesthetized with levamisole solution (60 µM). The *C. elegans* were pipetted onto a glass slide containing a 2% agarose pad. The autofluorescence images of *C. elegans* in each group were captured under a fluorescence microscope. The images were analyzed using the ImageJ V1.8.0 software, and the results were expressed as mean fluorescence intensity.

### 2.7. Assay of ROS

The ROS level in *C. elegans* was determined using the 2′,7′-dichlorodihydrofluorescein diacetate (H_2_DCFDA) fluorescent probe method, with fluorescence intensity reflecting the relative intracellular ROS content. After PM_2.5_ exposure, nematodes in each group were collected, washed three times with the sterile K-medium to remove surface-adhered particulates, and incubated with 10 μmol/L H_2_DCFDA at 20 °C in the dark for 2 h. Following three additional washes to remove excess probes, nematodes were immobilized with 60 μmol/L levamisole on 2% agarose pads. Fluorescence images of the intestinal region were captured with a fluorescence microscope (FITC filter), and the mean fluorescence intensity was quantified by ImageJ to represent the in vivo ROS level. At least 20–25 nematodes were tested per group, and all experiments were independently repeated three times.

### 2.8. Statistical Analysis

Three replicate experiments were performed for each exposure group. The results are presented as the mean ± standard error of the mean (SEM). Data analyses were performed using SPSS 26.0. The absolute value of the Pearson correlation coefficient was used to indicate the strength of the association, with a larger absolute value denoting a stronger correlation. A correlation was considered statistically significant at *p* < 0.05 and highly significant at *p* < 0.01. One-way analysis of variance (ANOVA) was applied for comparisons among multiple groups, followed by Dunnett’s test for post hoc comparisons between the control and each exposure group. Differences were deemed significant at *p* < 0.05 and highly significant at *p* < 0.01.

## 3. Results and Discussion

### 3.1. Chemical Characteristics of PM_2.5_

**Figure 1:**
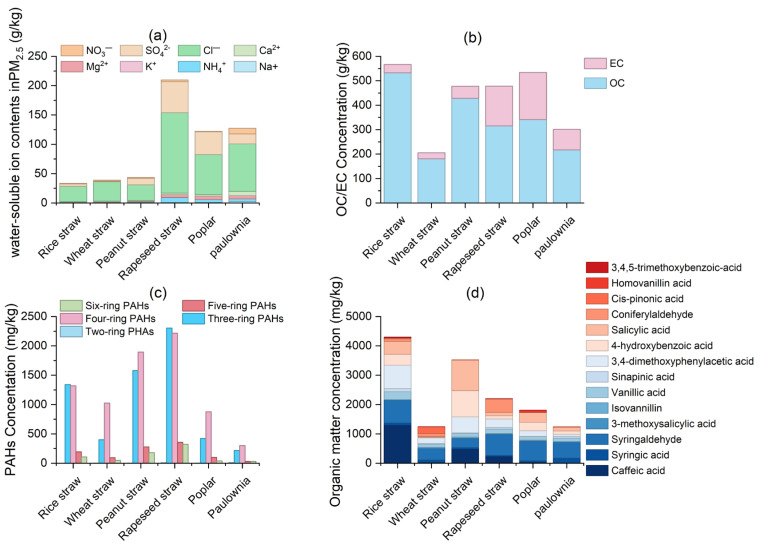
Chemical content of PM_2.5_ from various specific sources. The pictures show the (**a**) the mass of water-soluble ions contents (**b**) the OC/EC concentration (**c**) the polycyclic aromatic hydrocarbons concentration (**d**) the organic matter concentration in the PM_2.5_ from the combustion of various fuels.

The chemical composition of PM_2.5_ emitted from the incomplete combustion of various solid fuels shows significant characteristic difference (Figure 1). Detailed data can be found in Tables S1–S4 of the Supplementary Material. Carbonaceous aerosols account for a significant proportion of the total mass of the products of all fuels. As shown in Figure 1b, the particulate matter was overwhelmingly dominated by OC, with very low EC emissions. The exceptionally high OC/EC ratios observed are characteristic of low-temperature smoldering combustion processes. This extensive condensation of unburned and partially oxidized organic volatiles provides an abundant particulate carrier for toxic organic species.

The water-soluble inorganic ion profiles (Figure 1a) further distinguished the emission sources. As shown in the figure, the water-soluble ions in the combustion products of rapeseed straw have extremely high emission coefficients, exceeding 200 g per kilogram. These ions are mainly composed of chloride ions (Cl^−^) and potassium ions (K^+^), which are typical elemental markers of biomass combustion. In the combustion products of rapeseed straw and firewood, such as the branches of poplar and paulownia, these hygroscopic salts are abundantly loaded on the surface of the particles, which significantly alters the hygroscopicity of the particles. This enhanced hydrophilicity may facilitate the rapid dissolution and release of co-pollutants once the particles deposit into the physiological environment (e.g., the intestinal tract of *C. elegans*), thereby amplifying their bioavailability and acute toxicity.

**Figure 2:**
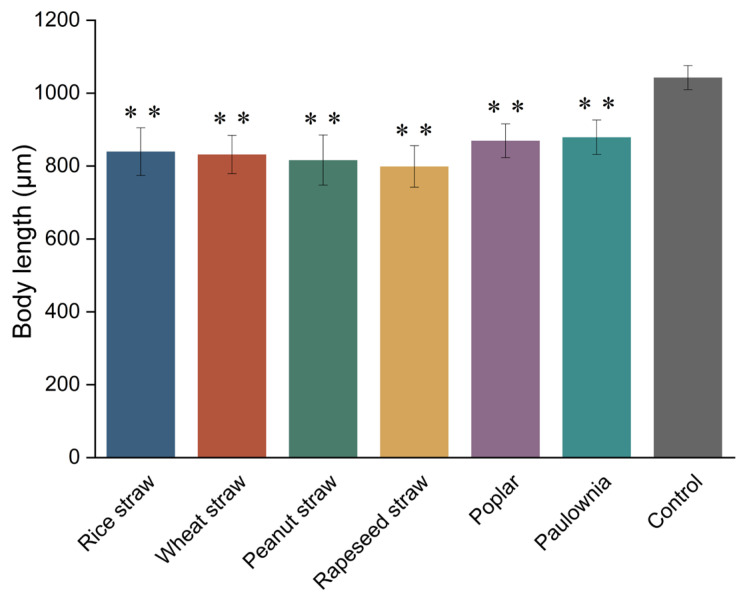
The impact of PM_2.5_ exposure on the body length of *C. elegans*. Significant differences between experimental and control groups are denoted by asterisks; ** indicates a highly significant difference (*p* < 0.01).

**Figure 3:**
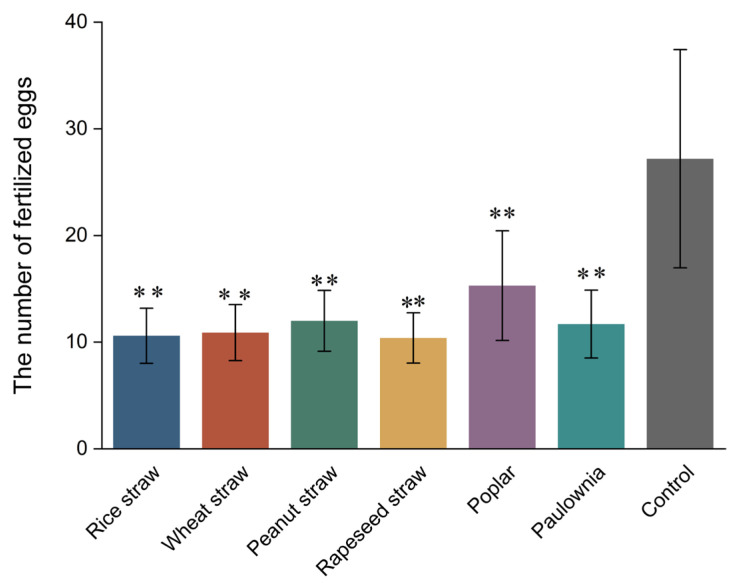
The impact of PM_2.5_ exposure on the number of fertilized eggs. Significant differences between experimental and control groups are denoted by asterisks; ** indicates a highly significant difference (*p* < 0.01).

The distribution of PAHs demonstrated a profound dependence on fuel type (Figure 1c), which serves as a critical chemical basis for the observed toxicological endpoints. The total emissions of PAHs from the combustion of oilseed crops, especially the rapeseed and peanut straws, are the highest, approximately 5200 milligrams per kilogram and 4000 milligrams per kilogram respectively, significantly exceeding those of grain crops and firewood. Driven by their high native lipid contents, the pyrolysis of oilseed crops during smoldering strongly favors the formation of complex, high-molecular-weight PAHs (e.g., benzo[a]pyrene, chrysene, and benzo[a]anthracene). These lipophilic and highly mutagenic PAHs can readily penetrate biological barriers. Their enrichment perfectly corroborates the severe developmental inhibition (Figure 2) and significant decline in fecundity (Figure 3) observed in nematodes exposed to oilseed crop-derived PM_2.5_, as these compounds are well-documented to trigger DNA damage and germline apoptosis.

In addition, specific organic biomarkers associated with lignin pyrolysis were quantitatively analyzed (Figure 1d). Significant concentrations of aldehydic species, such as coniferylaldehyde and syringaldehyde, alongside various methoxyphenols, were detected across the samples. Carbonyl compounds are highly electrophilic and are recognized as primary drivers of oxidative stress in biological systems. They have a significant potential synergistic effect with oxidoreduction-active phenolic substances and PAHs, providing chemical conditions for the generation of a large amount of reactive oxygen species and the accumulation of a large amount of lipofuscin in the exposed *C. elegans*.

### 3.2. Effect of PM_2.5_ on Development

A significant inhibition of *C. elegans* development (*p* < 0.001) was observed after exposure to PM_2.5_ from incomplete solid fuel burning. The specific body length data of *C. elegans* exposed to PM_2.5_ produced by incomplete combustion of different types of fuels are presented in Table S5 of the Supplementary Materials.

Specifically, the average body length of *C. elegans* in the exposure groups (839.6–878.8 μm) was reduced by 18.6% to 30.5% relative to the control (1042.6 μm), confirming the adverse effect of PM_2.5_ on growth. Notably, PM_2.5_ from oilseed crops, such as peanut and rapeseed, exhibited the most significant inhibitory effect. The impact of grain crops (e.g., rice and wheat) was generally more pronounced than firewood (e.g., poplar and paulownia).

Previous research has indicated that the differential toxicity among fuel types may be related to the distinct chemical profiles of emitted PM_2.5_. *C. elegans* have been widely employed as a model animal and demonstrated to be sensitive to PM_2.5_ exposure in toxicological research. Significant adverse effects on growth, reproduction, and locomotion have been consistently observed [23,24]. In the present study, component analysis revealed higher loads of PAHs in oilseed crop derived from PM_2.5_, which corresponds well with the observed strongest growth inhibition. Based on the existing literature, such components may interfere with growth-related signaling pathways such as the insulin/IGF-1 pathway via inducing oxidative stress, but the exact underlying pathway in this study remains to be further verified [25].

### 3.3. Effect of PM_2.5_ on the Number of Fertilized Eggs

To investigate the reproductive toxicity of PM_2.5_, the effect on the fecundity of *C. elegans* was evaluated in this study. The results indicated that exposure to PM_2.5_ generated by the combustion of different solid fuels significantly reduced the number of fertilized eggs in the uterus. The data of the number of fertilized eggs of *C. elegans* exposed to PM_2.5_ produced by incomplete combustion of different types of fuels are presented in Table S6. Specifically, the number of fertilized eggs in the exposure groups decreased by 43.8% to 61.8% compared with the control group. The most pronounced inhibitory effects were exhibited by PM_2.5_ from straw of oilseed crops (e.g., rapeseed) and grain crops (e.g., rice and wheat), with the average number of fertilized eggs reduced by more than half (e.g., a 61.8% reduction in the rapeseed PM_2.5_ exposure group). Although a significant inhibitory effect was also observed for PM_2.5_ from firewood (e.g., poplar), its adverse impact was generally less severe than that of the former two fuel types.

The inhibitory effect of PM_2.5_ on fecundity may be associated with germ cell damage induced by key toxic components such as PAHs, a mechanism that has been widely documented in previous toxicological studies [26]. These components have been shown to penetrate biological barriers and act directly on germ cells, inducing DNA double-strand breaks and other forms of damage. Such damage activates the highly conserved DNA damage checkpoint response (e.g., the HUS-1–CEP-1–p53 pathway) in *C. elegans*, thereby triggering the apoptotic program [27]. This leads to a large number of germline apoptosis during development, ultimately resulting in a sharp decline in the number of mature oocytes capable of successful fertilization. Furthermore, growing evidence has indicated that such environmental toxicants can also disrupt the epigenetic programming of germ cells (e.g., abnormal histone modification patterns). Although this effect may not directly induce apoptosis, it impairs the normal development and differentiation processes of germ cells, further compromising their fertilization and embryonic developmental potential at a functional level [28,29]. Thus, PM_2.5_ exposure significantly reduced fecundity, potentially through a dual mechanism involving the induction of genetic damage and interference with epigenetic regulation. Differences in toxicity among PM_2.5_ from various sources may be associated with variations in the content of key components. In line with chemical analysis results, the high lipid content of oilseed crops likely contributes to higher emissions of PAHs during incomplete combustion, which may account for the more severe reproductive toxicity observed in the present study.

### 3.4. Effect of PM_2.5_ on Lipofuscin

**Figure 4:**
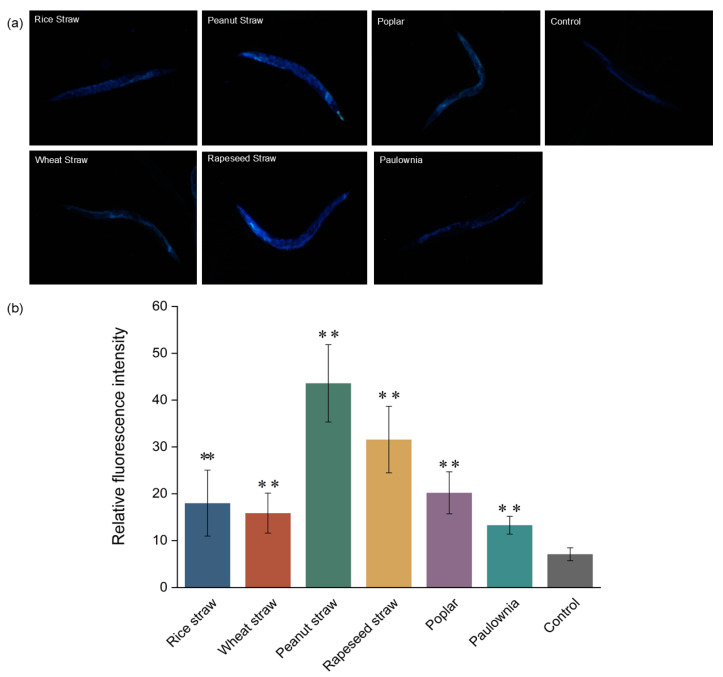
(**a**) The impact of PM_2.5_ exposure on lipofuscin production. The intensity of the blue fluorescence in the picture indicates the amount of lipofuscin accumulation. (**b**) Fluorescent image of lipofuscin. Significant differences between experimental and control groups are denoted by asterisks; ** indicates a highly significant difference (*p* < 0.01).

To assess the impact of PM_2.5_ on intestinal health, the level of intestinal autofluorescence (lipofuscin) was detected in this study to analyze the damage caused by PM_2.5_ from the combustion of different solid fuels to intestinal cells in *C. elegans*. Detailed data can be found in Table S7. The results indicated that exposure to PM_2.5_ from the straw of grain crops (e.g., rice and wheat) and firewood (e.g., poplar) did not induce a significant increase in lipofuscin levels in the nematodes, suggesting that the direct intestinal damage caused by these two types of PM_2.5_ was relatively limited. In contrast, exposure to PM_2.5_ generated from the combustion of oilseed crops (peanut and rapeseed) significantly elevated the lipofuscin levels in the nematodes (*p* < 0.001 for the peanut group; *p* < 0.01 for the rapeseed group), indicating that oilseed crop-derived PM_2.5_ induced a more pronounced increase in intestinal autofluorescence, reflecting more severe intestinal cell damage (Figure 4).

Lipofuscin, an accumulation of undegraded protein and lipid residues within lysosomes, is closely associated with dysfunction of organelles (particularly mitochondria and lysosomes) and disruption of proteostasis [30,31]. The more significant accumulation of lipofuscin induced by oilseed crop PM_2.5_ may be attributed to higher loads of toxic organic components in corresponding samples, which is supported by the chemical characterization results in this study. These components possess not only genotoxic potential but also the capacity to directly impair the function of organelles in intestinal epithelial cells.

Studies have shown that PAHs and their metabolites can intercalate into cell membranes, disrupt the mitochondrial electron transport chain, and lead to energy metabolism dysfunction and excessive ROS production. Concurrently, PAHs can directly inhibit lysosomal hydrolase activity or compromise lysosomal membrane integrity. This synergistic damage to mitochondria and lysosomes severely compromises the cell’s ability to clear metabolic waste, resulting in the substantial deposition of damaged proteins and lipids within lysosomes and the subsequent formation and accumulation of lipofuscin. In comparison, PM_2.5_ from grain crops and firewood contained lower loads of these key toxic organic components, which corresponds with the observation that no significant lipofuscin accumulation was induced by these samples. However, the exact subcellular mechanism underlying lipofuscin elevation in this study requires further direct verification.

### 3.5. Effect of PM_2.5_ on ROSs

**Figure 5:**
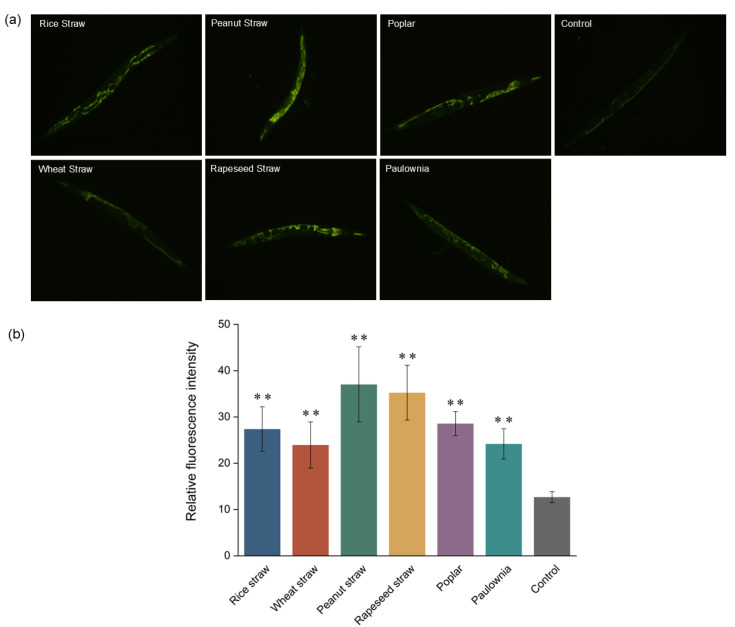
(**a**) The impact of PM_2.5_ exposure on ROS production. The intensity of the green fluorescence in the picture indicates the amount of ROS production. (**b**) Fluorescent image of ROS production. Significant differences between experimental and control groups are denoted by asterisks; ** indicates a highly significant difference (*p* < 0.01).

Oxidative stress is widely recognized as a core indicator for the toxicity evaluation of particulate matter (PM) [32,33]. The present study was designed to assess the oxidative stress induced by PM_2.5_ exposure. The analysis results are shown in Figure 5 and the detailed data can be found in Table S8. ROS levels in *C. elegans* were detected for quantitative analysis, and toxicity differences in PM_2.5_ from solid fuel combustion sources were systematically compared. Analytical results indicated that exposure to PM_2.5_ generated from the combustion of multiple-solid fuel significantly elevated intestinal ROS levels in *C. elegans*. PM_2.5_ derived from oilseed crops, including peanut and rapeseed, induced a highly significant increase in ROS levels (*p* < 0.001), accompanied by the most substantial accumulation of lipofuscin, confirming the most severe impact on the *C. elegans* antioxidant defense system. In contrast, a significant elevation in ROS levels (*p* < 0.05) was also induced by PM_2.5_ derived from the combustion of grain crops and firewood, with adverse effect intensities confirmed to be weaker than the effect intensities induced by oilseed crop-derived PM_2.5_.

Disruption of systemic redox homeostasis was identified as the core pathogenesis, mediated by the size effect and combined toxicities of multiple chemical components in PM_2.5_ [34,35]. PM_2.5_ was confirmed to penetrate the intestinal epithelial barrier and accumulate in intestinal tissues of *C. elegans*, providing a structural basis for toxic effect exertion [19,36,37]. Multiple components in PM_2.5_, including PAHs and carbonaceous fractions, were verified to synergistically trigger ROS overproduction via activation of metabolic redox cycling, mitochondrial function impairment, Fenton-like reactions, and endogenous antioxidant system depletion [4,13,38,39]. Redox imbalance was ultimately induced, with corresponding ROS elevation and lipofuscin accumulation in exposed *C. elegans*.

The differential capacity of toxic effects of PM_2.5_ emitted from the combustion of different solid fuels were primarily attributed to fuel-specific chemical composition and the transformation products formed during the combustion process. Consistent with the measurement results, oilseed crop combustion generates higher concentrations of lipophilic PAHs and their derivatives. For PAHs, it was confirmed that far stronger toxicity to nematodes was exerted by high-molecular-weight, high-toxicity PAHs such as benzo[a]pyrene than by low-molecular-weight PAHs, with a 72 h LC_50_ that was two orders of magnitude lower than the value recorded for low-ring PAHs [40,41]. In the present investigation, the strongest induction of intestinal ROS and lipofuscin in *C. elegans* by PM_2.5_ from oilseed crops (e.g., peanut, rapeseed) was associated with the potentially higher core toxic loads of oilseed crop-derived particulate matter. Additionally, it was revealed that environmentally persistent free radicals (EPFRs), which are commonly generated during incomplete solid fuel combustion, could serve as another critical toxic component. Such environmentally persistent free radicals were found to persist in particulate matter for an extended period and continuously induce ROS generation, and the toxicity of combustion-derived PM_2.5_ was proven to decrease synchronously with the attenuation of such persistent radicals [39]. Previous studies revealed that the ROS level induced by PM_2.5_ in nematodes was positively correlated with the exposure concentration of these toxic components, and the magnitude of ROS elevation directly corresponded to the toxicity intensity [42]. Therefore, although significant ROS induction was also detected in *C. elegans* exposed to cereal and fuelwood-derived PM_2.5_, the lower content of key toxic components resulted in a smaller magnitude of ROS elevation and weaker synergistic toxic effects, which ultimately led to weaker overall adverse effects compared with those induced by oilseed crop-derived PM_2.5_.

## 4. Conclusions

The present study was designed to investigate the chemical characteristics and toxic effects of PM_2.5_ emitted from incomplete combustion of six common solid fuels, and to elucidate the underlying toxicological mechanisms using *C. elegans* as a model organism. Chemical compositions of PM_2.5_ were found to be significantly source-dependent, with carbonaceous aerosols dominated by organic carbon (OC) constituting the major fraction across all fuel types. High OC/EC ratios characteristic of low-temperature smoldering combustion were observed in all samples.

Rapeseed straw exhibited the highest emission factor of water-soluble ions (exceeding 200 g/kg), overwhelmingly dominated by chloride and potassium. Oilseed crops (peanut and rapeseed straws) generated the highest total PAH emissions (approximately 5200 mg/kg and 4000 mg/kg, respectively), significantly surpassing those from grain crops and fuelwood. Significant concentrations of aldehydic species and methoxyphenols were also detected across all samples.

PM_2.5_ from incomplete solid fuel burning exerted significant toxic effects on *C. elegans*, including developmental inhibition, reproductive damage, intestinal injury, and oxidative stress. The average body length of exposed nematodes was reduced by 18.6% to 30.5% relative to the control, and the number of fertilized eggs decreased by 43.8% to 61.8%. Oilseed crop-derived PM_2.5_ exhibited the most severe toxic effects, as evidenced by the greatest reduction in body length and fertilized egg number, the most significant accumulation of lipofuscin, and the highest level of ROS production. Toxic effects of cereal crop-derived PM_2.5_ were generally more pronounced than those of firewood-derived PM_2.5_.

Severe oxidative stress and ROS overproduction were identified as the primary toxicological mechanisms of PM_2.5_ from incomplete solid fuel burning. High loads of lipid-soluble PAHs in PM_2.5_ synergistically induced ROS overproduction. The present study clarifies source-dependent differences in chemical characteristics and toxic effects of PM_2.5_ from incomplete solid fuel burning and reveals the core toxicological mechanism mediated by oxidative stress. Findings from this study provide important experimental data for understanding the environmental risks of solid fuel combustion-derived PM_2.5_ and offer a theoretical basis for the development of targeted pollution control measures. While further studies are needed to investigate long-term and transgenerational toxic effects of these particles, the results presented here highlight the necessity of prioritizing control of oilseed crop straw burning to reduce PM_2.5_-related health risks.

Some limitations of this study should be noted. A single exposure concentration was adopted for comparative toxicological evaluation, which cannot fully characterize the dose–response relationship of PM_2.5_ from different sources, nor can it accurately quantify the relative toxicity potency and toxic threshold of particulate matter from each source. Whether oilseed crop-derived PM_2.5_ maintains higher toxicity at lower exposure concentrations remains to be verified by systematic multi-concentration gradient experiments.

## Data Availability

The original contributions presented in this study are included in the article/Supplementary Materials. Further inquiries can be directed to the corresponding authors.

## Supplementary Materials

The following supporting information can be downloaded at https://www.mdpi.com/article/10.3390/toxics14070597/s1, Table S1: Mass concentration of water-soluble ions in PM_2.5_ samples (g/kg); Table S2: Mass concentration of OC/EC in PM_2.5_ samples (g/kg); Table S3: Mass concentration of PAHs in PM_2.5_ samples (mg/kg); Table S4: Mass concentration of organic biomarkers associated with lignin pyrolysis in PM_2.5_ samples (mg/kg); Table S5: Body length of *C. elegans* after exposure to PM_2.5_ from incomplete combustion of different types of solid fuel; Table S6: Numbers of fertilized eggs in *C. elegans* after exposure to PM_2.5_ from incomplete combustion of different types of solid fuel; Table S7: Relative fluorescence intensity of lipofuscin in *C. elegans* after exposure to PM_2.5_ from incomplete combustion of different types of solid fuel; Table S8: Relative fluorescence intensity caused by the generation of ROS in *C. elegans* after exposure to PM_2.5_ from incomplete combustion of different types of solid fuels.
