# Supplementary material for: Toxicity Effects of Fine Particulate Matter (PM2.5) from Incomplete Solid Fuel Burning in Caenorhabditis elegans
Source: Toxics. 2026 Jul 8;14(7):597. doi: 10.3390/toxics14070597 (PMC13417411; doi:10.3390/toxics14070597)
Supplement: Supplementary file 1 [file toxics-14-00597-s001.zip › toxics-4364871-supplementary.pdf]

Supplementary Material for

Toxicity Effects of Fine Particulate Matter ($PM_{2.5}$) from Incomplete Solid Fuel Burning in *Caenorhabditis elegans*

Zhenyu Lu [1], Bingbo Huang [1], Xiaoming Liu [2,*], Wankang Chen [3], Xiaoyu Cai [1] and Mindong Chen [1,*]

[1] Collaborative Innovation Center of Atmospheric Environment and Equipment Technology, Jiangsu Key Laboratory of Atmospheric Environment Monitoring and Pollution Control, School of Environmental Science and Engineering, Nanjing University of Information Science & Technology,
Nanjing 210044, China; nuistlzy@163.com (Z.L.); 13182805780@163.com (B.H.); caixiaoyu8410@163.com (X.C.)

[2] Jiangsu Environmental Engineering Technology Co., Ltd., Nanjing 210044, China

[3] School of Environment, Southern University of Science and Technology, Shenzhen 518055, China; 12531399@mail.sustech.edu.cn

* Correspondence: l17864182144@163.com (X.L.); chenmd@nuist.edu.cn (M.C.)

Table S1 Mass concentration of water-soluble ions in $PM_{2.5}$ samples (g/kg)

Water-soluble ions	Rice straw	Wheat straw	Peanut straw	Rapeseed straw	Poplar	Paulownia
Na^+	46.89	58.67	79.42	71.27	98.08	257.43
NH_4^+	43.66	30.83	106.14	823.98	466.16	454.56
K^+	18.46	29.80	14.02	79.47	35.20	0.42
Mg^{2+}	45.27	68.13	111.79	443.74	568.60	538.80
Ca^{2+}	74.37	132.47	135.80	251.24	264.73	735.93
CI^-	2623.64	3292.08	2657.62	13720.44	6811.77	8090.85
SO_4^{2-}	394.87	99.56	1119.42	5272.82	3880.33	1695.78
NO_3^-	99.45	191.75	142.50	303.24	89.48	974.72
Total	46.89	58.67	79.42	71.27	98.08	257.43

Table S2 Mass concentration of OC/EC in $PM_{2.5}$ samples (g/kg)

Fuels	OC	EC
Rice straw	532.25	34.48
Wheat straw	180.54	24.68
Peanut straw	428.50	48.73
Rapeseed straw	315.15	162.76
Poplar	341.33	192.52
Paulownia	216.90	84.14

Table S3 Mass concentration of PAHs in $PM_{2.5}$ samples (mg/kg)

PAHs	Rice straw	Wheat straw	Peanut straw	Rapeseed straw	Poppy branches	Sycamore branches
NAP	0.94	2.07	2.44	8.13	2.32	11.54
ACE	37.77	5.22	3.98	4.87	7.33	26.66
ACY	21.87	3.43	31.77	39.35	7.31	10.56
FLO	246.62	6.83	95.98	144.43	15.23	29.08
PHE	488.20	287.77	941.35	1392.02	295.53	115.54
ANT	545.33	97.88	507.05	723.23	97.78	35.39
FLA	528.36	308.79	727.17	934.83	283.85	100.90
PYR	547.65	363.48	707.87	890.55	317.27	166.91
BaA	182.71	277.60	268.23	303.23	167.13	20.97
CHR	61.21	75.71	191.83	86.40	106.95	13.81
Bb&KF	88.31	49.00	125.62	168.24	55.85	13.35
BaP	90.61	40.41	125.50	166.06	38.24	13.82
IcdP	57.86	25.12	97.92	162.17	22.03	15.02
BghiP	50.48	22.20	83.41	159.67	17.85	16.53
DahA	14.86	5.53	25.70	21.33	5.97	3.91

Table S4 Mass concentration of organic biomarkers associated with lignin pyrolysis in $PM_{2.5}$ samples (mg/kg)

Organic Matter	Rice straw	Wheat straw	Peanut straw	Rapeseed straw	Poplar	Paulownia
Caffeic acid	1292.27	27.20	477.88	227.50	34.63	0.08
Syringic acid	75.42	82.91	51.13	38.03	49.55	176.59
Syringaldehyde	784.88	384.68	319.77	728.55	665.30	527.34
3-methoxysalicylic acid	13.44	45.92	7.04	NF	12.99	35.65
Isovannillin	NF	NF	36.81	19.13	24.92	NF
Vanillic acid	270.16	104.44	124.74	147.61	118.10	112.34
Sinapinic acid	109.36	28.45	15.00	56.98	9.62	61.75
3,4-dimethoxyphenylacetic acid	794.87	190.22	544.64	285.89	190.54	70.62
4-hydroxybenzoic acid	376.03	21.40	897.16	112.90	290.45	111.70
Salicylic acid	436.88	22.41	1035.41	119.51	327.40	122.07
Coniferylaldehyde	83.58	106.75	21.21	453.42	16.67	32.17
Cis-pinonic acid	31.19	225.33	NF	NF	NF	NF
Homovanillin acid	21.93	NF	NF	13.50	67.39	0.00
3,4,5-trimethoxybenzoic-acid	9.90	4.09	NF	0.00	0.65	0.00

Table S5 Body length of *C. elegans* after exposure to $PM_{2.5}$ from incomplete combustion of different types of solid fuel

Types of solid fuels		Body length	
		Mean	Standard Deviation
Grain Crop	Rice straw	839.59	65.34
	Wheat straw	831.66	52.46
Oil Crop	Peanut straw	816.34	68.71
	Rapeseed straw	798.81	56.83
Fire wood	Poplar	869.33	46.47
	Paulownia	878.81	47.25
Control		1042.63	33.14

Table S6 Numbers of fertilized eggs in *C. elegans* after exposure to $PM_{2.5}$ from incomplete combustion of different types of solid fuel

Fuels	Numbers of fertilized eggs	
	Mean	Standard Deviation
Rice straw	10.55	2.58
Wheat straw	10.88	2.63
Peanut straw	12.04	2.85
Rapeseed straw	10.38	2.36
Poplar	15.30	5.14
Paulownia	11.73	3.18
Control	27.23	10.23

Table S7 Relative fluorescence intensity of lipofuscin in *C. elegans* after exposure to $PM_{2.5}$ from incomplete combustion of different types of solid fuel

Types of solid fuels		Relative fluorescence intensity	
		Mean	Standard Deviation
Grain Crop	Rice straw	17.98	7.04
	Wheat straw	15.86	4.27
Oil Crop	Peanut straw	43.58	8.26
	Rapeseed straw	31.56	7.11
Fire wood	Poplar	20.20	4.49
	Paulownia	13.27	1.91
Control		7.07	1.39

Table S8 Relative fluorescence intensity caused by the generation of ROS in *C. elegans* after exposure to $PM_{2.5}$ from incomplete combustion of different types of solid fuels

Types of solid fuels		Relative fluorescence intensity	
		Mean	Standard Deviation
Grain Crop	Rice straw	27.39	4.82
	Wheat straw	23.95	4.99
Oil Crop	Peanut straw	37.02	8.11
	Rapeseed straw	35.24	5.93
Fire wood	Poplar	28.60	2.6
	Paulownia	24.19	3.26
Control		12.67	1.19
